# Supplementary material for: Spin-orbit-splitting-driven nonlinear Hall effect in NbIrTe4
Source: Nat Commun. 2024 May 10;15:3971. doi: 10.1038/s41467-024-47643-4 (PMC11087648; doi:10.1038/s41467-024-47643-4)
Supplement: Supplementary file 1 — Supplementary Information [file 41467_2024_47643_MOESM1_ESM.docx]

Supplementary Information for

**Spin-orbit-splitting-driven nonlinear Hall effect in NbIrTe­_4_**

Ji-Eun Lee^1,2,3,4^, Aifeng Wang^5,6^, Shuzhang Chen^5,7^, Minseong Kwon^2,8^, Jinwoong Hwang^1,9^, Minhyun Cho^8^, Ki-Hoon Son^2^, Dong-Soo Han^2^, Jun Woo Choi^2^, Young Duck Kim^8^, Sung-Kwan Mo^1^, Cedomir Petrovic^5,7,10^, Choongyu Hwang^3,*^, Se Young Park^11,12,*^, Chaun Jang^2,*^, and Hyejin Ryu^2,*^

^1^Advanced Light Source, Lawrence Berkeley National Laboratory, Berkeley, CA 94720, USA

^2^Center for Spintronics, Korea Institute of Science and Technology (KIST), Seoul 02792, South Korea

^3^Department of Physics, Pusan National University, Busan 46241, South Korea

^4^Max Planck POSTECH Center for Complex Phase Materials, Pohang University of Science and Technology, Pohang 37673, South Korea

^5^Condensed Matter Physics and Materials Science Department, Brookhaven National Laboratory, Upton, New York 11973, United States

^6^Low Temperature Physics Laboratory, College of Physics and Center of Quantum Materials and Devices, Chongqing University, Chongqing 400044, China

^7^Department of Physics and Astronomy, Stony Brook University, Stony Brook, New York 11794-3800, USA

^8^Department of Physics and Department of Information Display, Kyung Hee University, Seoul 02447, South Korea

^9^Department of Physics and Institute of Quantum Convergence Technology, Kangwon National University, Chuncheon 24341, South Korea

^10^Shanghai Advanced Research in Physical Sciences, Shanghai 201203, China

^11^Department of Physics and Origin of Matter and Evolution of Galaxies (OMEG) Institute, Soongsil University, Seoul 06978, South Korea

^12^Integrative Institute of Basic Sciences, Soongsil University, Seoul, 06978, South Korea

*e-mail: [ckhwang@pusan.ac.kr](mailto:ckhwang@pusan.ac.kr), [sp2829@ssu.ac.kr](mailto:sp2829@ssu.ac.kr), [cujang@kist.re.kr](mailto:cujang@kist.re.kr), [hryu@kist.re.kr](mailto:hryu@kist.re.kr)

**Supplementary Note 1. First harmonic Hall voltage of NbIrTe_4_**


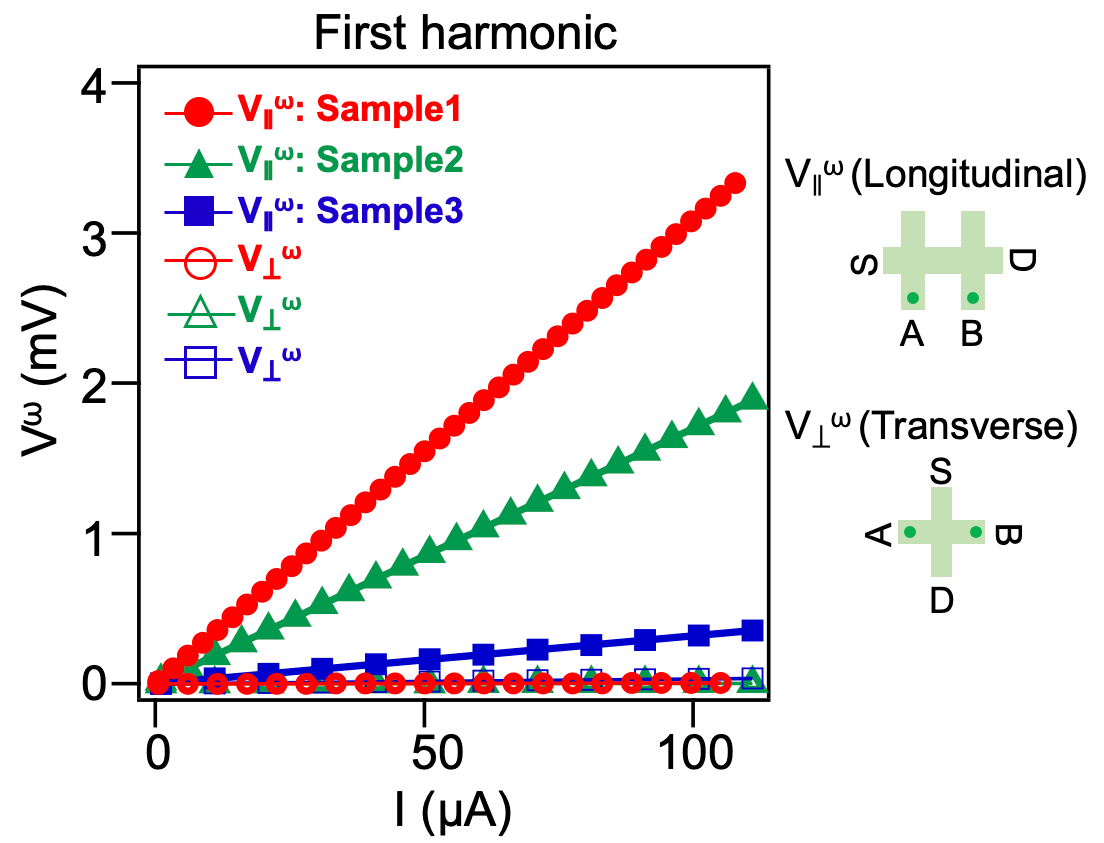


**Supplementary Fig. 1**. First-harmonic $V_{\parallel}^{\omega}$ (longitudinal) and $V_{\perp}^{\omega}$ (transverse) as function of the AC current I ^ω^ for three different samples at 2 K. (Sample 1, Sample 2, and Sample 3). All measured $V_{\parallel}^{\omega}$values (closed symbols) consistently exceed the corresponding *V*_⊥_^ω^ values (open symbols), indicating the disappearance of the linear Hall response ($V_{\perp}^{\omega}$) due to preserved time-reversal symmetry. The green crossbars illustrate the experimental geometries. $V_{\parallel}^{\omega}$ and $V_{\perp}^{\omega}$ represent the first-harmonic voltage parallel and perpendicular to the applied current direction (four probe *IV* measurement and Hall measurement), respectively. The applied current is injected from the source (S) electrode to the drain (D) and the voltage is measured between A and B electrodes.

First harmonic voltages of NbIrTe_4_ are measured for three different flakes at 2 K. The longitudinal voltage $V_{\parallel}^{\omega}$ responds linearly with the current$I^{\omega}$ along the *a*-axis, identical to the behavior observed in the four-probe *I*–*V* measurement result, whereas the transverse voltage$V_{\perp}^{\omega}$ (Hall voltage) is much smaller than $V_{\parallel}^{\omega}$ across all three samples. Due to the preserved time-reversal symmetry, the linear Hall response ($V_{\perp}^{\omega}$) should vanish due to the zero BC nature^1^.

**Supplementary Note 2. The direction and frequency of driving AC current-dependent second harmonic Hall voltage of NbIrTe_4_**


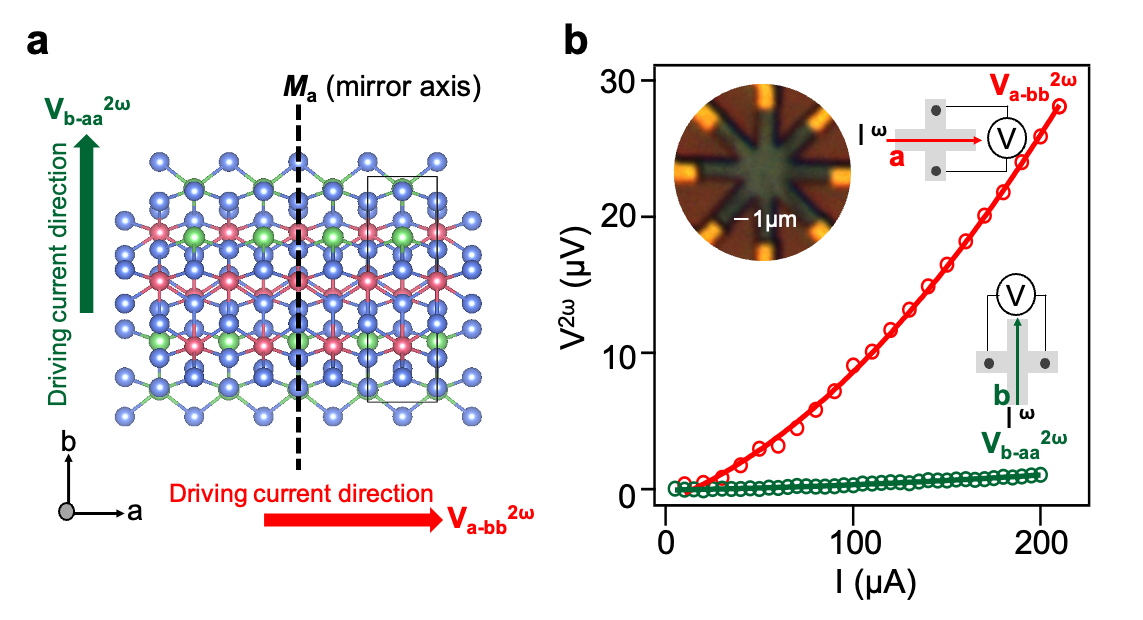


Supplementary Fig. 2. The direction of driving AC current direction-dependent nonlinear Hall voltage ($\boldsymbol{V}^{\boldsymbol{2}\boldsymbol{\omega}}$) in NbIrTe_4_. (a) The in-plane crystal structure with unit cell of square box. The mirror axis is parallel to *b*-axis and perpendicular to *a*-axis. The AC current is applied along *a*- and *b*-axis, labeled as $\boldsymbol{V}_{\boldsymbol{a-bb}}^{\boldsymbol{2}\boldsymbol{\omega}}$ and $\boldsymbol{V}_{\boldsymbol{b-aa}}^{\boldsymbol{2}\boldsymbol{\omega}}$. (b) The second-harmonic transverse voltage depending on driving current direction along the different two crystal axes. The inset shows an optical microscopic image of the measured device references with 1 µm scale bars for reference.

We measured second harmonic transverse Hall voltage as varying current direction along the two different crystal axes i.e., a-axis and b-axis in Supplementary Fig. 2. Considering crystal symmetry, only the driving current along a-axis should exhibit the NLHE signal. Evidently, the second-harmonic Hall voltage ($V_{a-bb}^{2\omega}$) with driving current along the a-axis exceedingly surpass the second-harmonic Hall voltage ($V_{b-aa}^{2\omega}$) with driving current along the b-axis, suggesting that there is the absence of NLHE along b-axis.


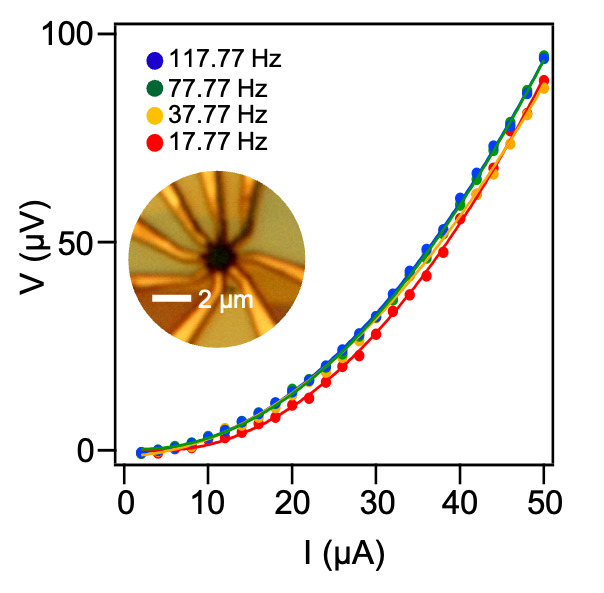


Supplementary Fig. 3. Nonlinear Hall voltage ($\boldsymbol{V}^{\boldsymbol{2}\boldsymbol{\omega}}$) for several different frequencies of driving AC current in NbIrTe_4_. The second-harmonic voltage for different driving current frequencies of 117.77 Hz, 77.77 Hz, 37.77 Hz, and 17.77 Hz. The inset shows an optical microscopic image of the measured device with a 1 µm scale bar.

We also measured second harmonic transverse Hall voltage by changing frequencies in Supplementary Fig. 3. We clearly observe no frequency dependence in the measurement, suggesting that we can exclude measurement artifacts.

**Supplementary Note 3. Nonlinear Hall strength of NbIrTe_4_ as a function of temperature**


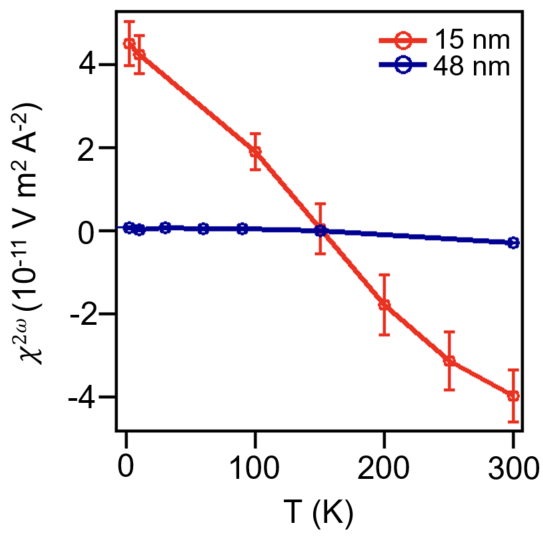


**Supplementary Fig. 4.** The strength of NLHE at different thickness of flakes, 15 nm and 48 nm, as a function of temperature.

We performed NLHE measurements on NbIrTe_4_ flakes with two different thicknesses, 15 nm and 48 nm, to compare the NLHE in different thickness. The strength of the nonlinear Hall conductivity, $\chi^{2\omega}$, indicates a quadratic coefficient of the parabolic fitting function of $V_{\perp}^{2\omega}$ vs. $I^{\omega}$. The sign of the NLH signal changes at ~150 K irrespective of the sample thickness, whereas the NLH signal $\chi^{2\omega}$ for 15 nm-thick flakes is substantially higher than that for 48 nm-thick flakes, supporting the previous argument that the NLH signal can only originate from the surface because of the absence of the glide mirror plane on the surface^2^.

**Supplementary Note 4. Determination of the thickness of the exfoliated NbIrTe_4_ flake by atomic force microscopy (AFM)**

**
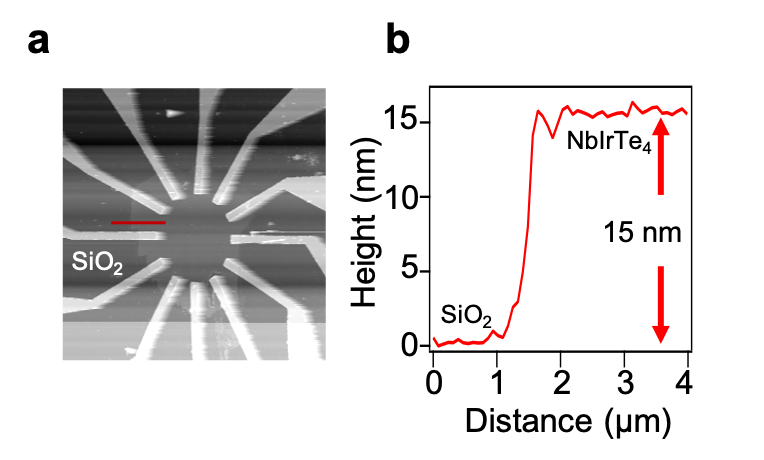
**

Supplementary Fig. 5. (a) An AFM image showing a 15 nm-thick flake of NbIrTe_4_ on an SiO_2_ substrate. (b) The measured height profile along the red line in panel a.

We confirmed the thickness of an exfoliated NbIrTe_4_ using atomic force microscopy (AFM). The AFM image is shown in Supplementary Fig. 5 (a), revealing the 15 nm-thick of NbIrTe_4_ flake on SiO_2_ substrate. The height profile of the NbIrTe_4_ flake in Supplementary Fig. 5 (b) was taken along the red line in (a).

**Supplementary Note 5. Temperature-dependent electronic band structures of NbIrTe_4_**


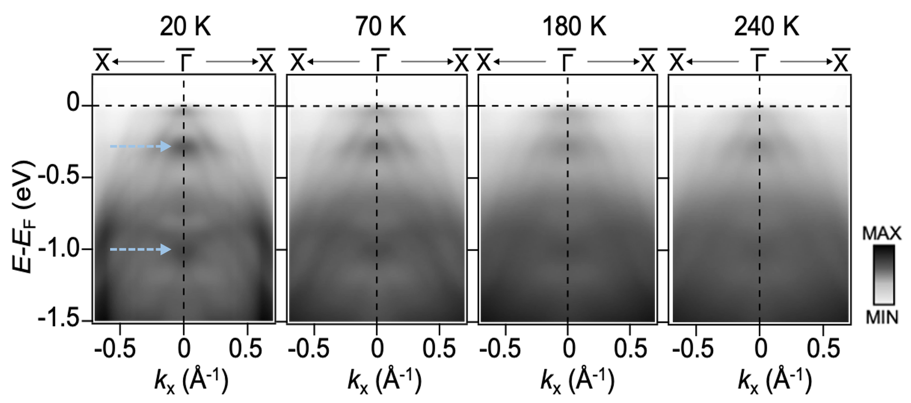


Supplementary Fig. 6. Temperature-dependent ARPES intensity cuts along the $\overline{X}$-$\overline{\Gamma}$-$\overline{X}$direction at temperature of 20, 70, 180, 240 K, respectively. Three horizontal arrows indicate the position of the three EDC peaks at *E*-*E*_F_ = -0.3 and -1.0 eV, respectively.

We show representative temperature-dependent ARPES *E*-*k* cuts along $\overline{\Gamma}$-$\overline{X}$ direction at temperature of 20 K, 70 K, 180 K, and 240 K, respectively. In Fig. 3(d), EDCs are extracted from the black dashed line at $\overline{\boldsymbol{\Gamma}}$ point in Supplementary Fig. 6.

**Supplementary Note 6. Multi-peak fit analysis of temperature-dependent EDCs**


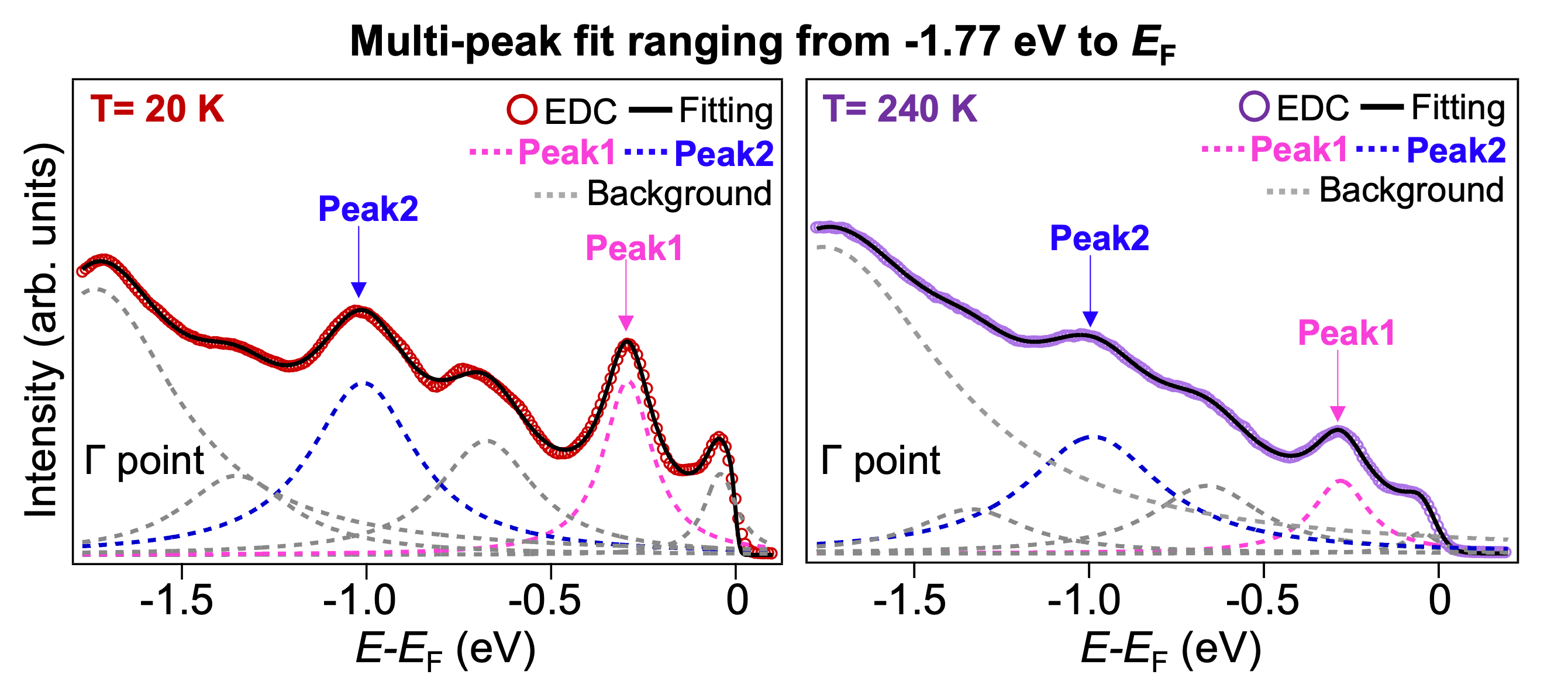
 **Supplementary Fig. 7.** Multi-peak fits of energy distribution curves (EDCs) at the $\overline{\boldsymbol{\Gamma}}$, which is fitted with Lorentzian peaks, incorporating the multiplication of the Fermi-Dirac distribution (FD) function and convolution with instrumental resolution. The energy range of fitting is from -1.77 eV to Fermi energy (*E*_F_) with temperatures ranging from 20 K to 240 K.

Our fitting analysis involves the Lorentzian peaks to cover a wide range of energy in the $\overline{\boldsymbol{\Gamma}}$ point, which is achieved by the multiplying the FD function and convoluting the instrumental resolution. Now, the fitted curves are accurately aligned with the Fermi level cut-off energy, as presented in Supplementary Fig. 7. Furthermore, when the temperature increases to 240 K ($\overline{\boldsymbol{\Gamma}}$ point), distinct peaks labeled as Peaks 1 and 2 ($\overline{\boldsymbol{\Gamma}}$ point) are clearly resolved. We note that other peaks denoted with grey dotted lines in Supplementary Fig. 7 exhibit significant broadening at higher temperatures, displaying fluctuating weight during the fitting process, rendering them unreliable.

**Supplementary Note 7. Thickness dependence of the Berry curvature dipole**

**Supplementary Fig. 8.** The calculated Berry curvature dipoles for supercells with various thicknesses of 8, 10, and 12 layers.

As the Berry curvature dipole (BCD) is calculated in a slab geometry with a thickness smaller than the thickness of an experimental sample, we checked the dependence of the calculated BCD as a function of slab thickness. Supplementary Fig. 8 shows the BCD calculated for slabs thicknesses of 8, 10, and 12 layers; further increasing the number of slabs is challenging because of the large numbers of Wannier functions required for calculating the BCD (1056 Wannier functions for 12 layers). We find that the key feature in the BCD (i.e., a positive-to-negative sign change as the chemical potential is increased) is maintained with increasing thickness. Thus, we expect that the same feature will be maintained for the thicker slab.

**Supplementary Note 8. Obtaining Berry curvature dipole from the transport data**

We estimate the BCD using the following formula from Eq (1):

|  | $j_{b}\left( 2\omega\right)=\frac{e^{3}\tau}{2\hbar^{2}\left( 1+i\omega\tau\right)}D_{a}{\left( \omega\right)\left\vert E_{a}\left( \omega\right) \right\vert}^{2}$, | (1) |
| --- | --- | --- |

where ℏ is Planck’s constant, *τ* is the scattering time, E*_a_*(*ω*) is an external electric field along the *a*-axis, *D_a_*(*ω*) is the BCD along the *a*-axis, and *j­­_b_*(2*ω*) is the second-harmonic current density along the *b*-axis. We assume the static limit (*ωτ* << 1)^3^ and express *D_a_*(*ω*) as

|  | $D_{a}\left( \omega\right)=\frac{2\hbar^{2}j_{b}\left( 2\omega\right)}{e^{3}\tau\left\vert E_{a}\left( \omega\right) \right\vert^{2}}=\frac{2\hbar^{2}nt^{2}WV_{b}\left( 2\omega\right)}{e {m^{*}\rho_{0}^{2} \left\vert I_{a}\left( \omega\right) \right\vert}^{2}}$, | (2) |
| --- | --- | --- |

where *n* is the charge density, *t* and *W* are the thickness and width of the sample, respectively, *V_b_*(2*ω*) is the second-harmonic Hall voltage along the *b*-axis, $m^{*}$ is the effective mass that we approximate as the electron mass (*m*_e_), *I_a_*(*ω*) is the current along the *a*-axis, and *ρ*_0_ is the resistivity in the static limit, given as $\frac{m^{*}}{ne^{2}\tau}$. By substituting *n* = 2.1 × 10^20^ cm^−3^, *t* = 3 × 10^−6^ cm, *W* = 5 × 10^−7^ cm, *V_b_*(2*ω*) = 10 μV, *ρ*_0_ = 30 μΩ cm, and *I_a_*(*ω*) = 60 μA, we obtain *D_a_*(*ω*) = 4.43. Since the BCD in the two-dimensional limit is compared between theory and experiments, the relevant value is *D_a_*(*ω*) × *t*_calc_ = 348 Å, where *t*_calc_ is the thickness of the 12-layer supercell (78.5 Å).

**Supplementary Note 9. Comparison of slab band structures of supercell tight-binding and DFT calculations**


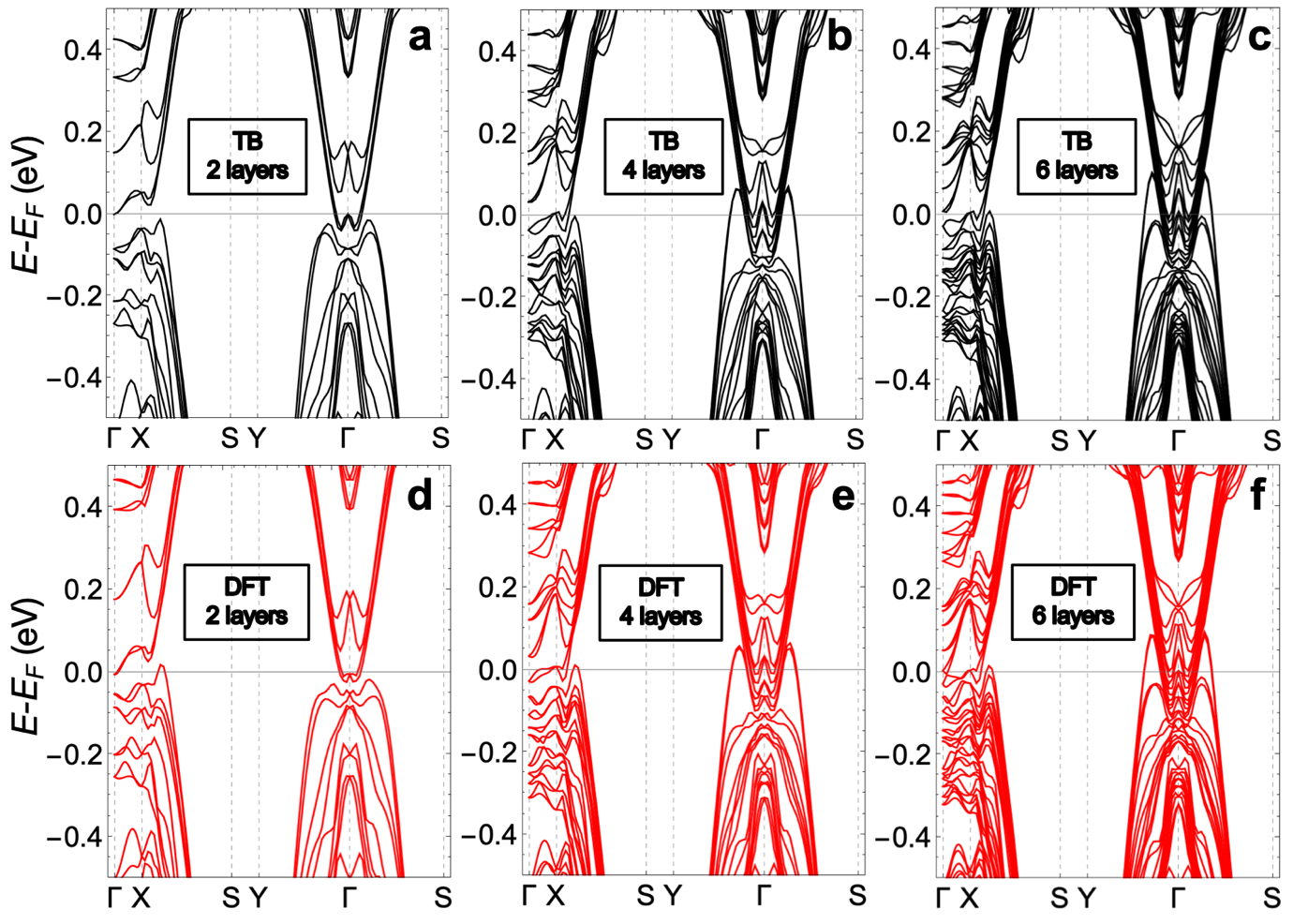


**Supplementary Fig. 9.** Comparison of slab band structures of supercell tight-binding (TB) and DFT calculations. The panel (a), (b), and (c) are TB band structures of two, four, and six-layer slabs calculated from supercell TB Hamiltonian, respectively, constructed from the bulk TB parameters. The panel (d), (e), and (f) are DFT band structures of two, four, and six-layer slabs, respectively, calculated with vacuum-slab geometry.

**Supplementary Note 10. Comparison of the band structures, BCD, and momentum-resolved BC between the GGA and LDA schemes**


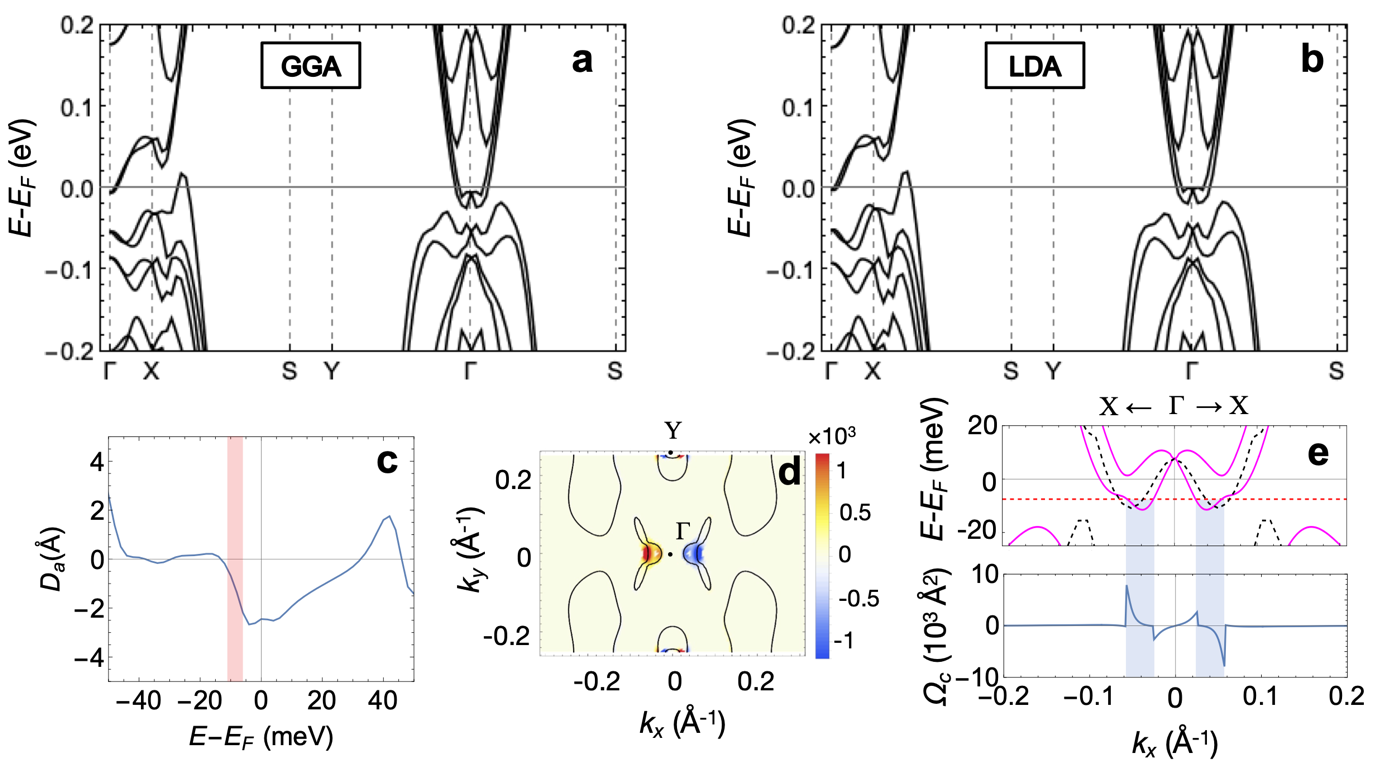


**Supplementary Fig. 10.** Comparison of BCD and momentum-resolved BC of the bilayer slab between LDA and GGA exchange-correlation functionals. **a-b.** Band structures along high-symmetry line calculated with GGA (panel a) and LDA (panel b) exchange-correlation functionals. The results in panels **c-e** are calculated with LDA. **c.** The BCD (*D_a_*) contributing to the in-plane NLHE for bilayer NbIrTe_4_, plotted as a function of the chemical potential. **d.** Momentum-resolved BC (*Ω_c_*(**k**) in Å^2^) of bilayer NbIrTe_4_ integrated from −11 to −6 meV corresponding to the red shaded area in panel c. **e.** Band dispersion around the *k*-points contributing large Berry curvatures of bilayer NbIrTe_4_. The top panel shows bands near the Fermi energy expressed as solid magenta lines along the X–Γ–X cut. The black dashed lines are the bands calculated without spin-orbit coupling. The bottom panel is BC (*Ω_c_*(**k**)) integrated up to −7.5 meV relative to *E_F_* (red dashed line).

**Supplementary Note 11. Determination of the surface termination and doping-dependent surface band structures**


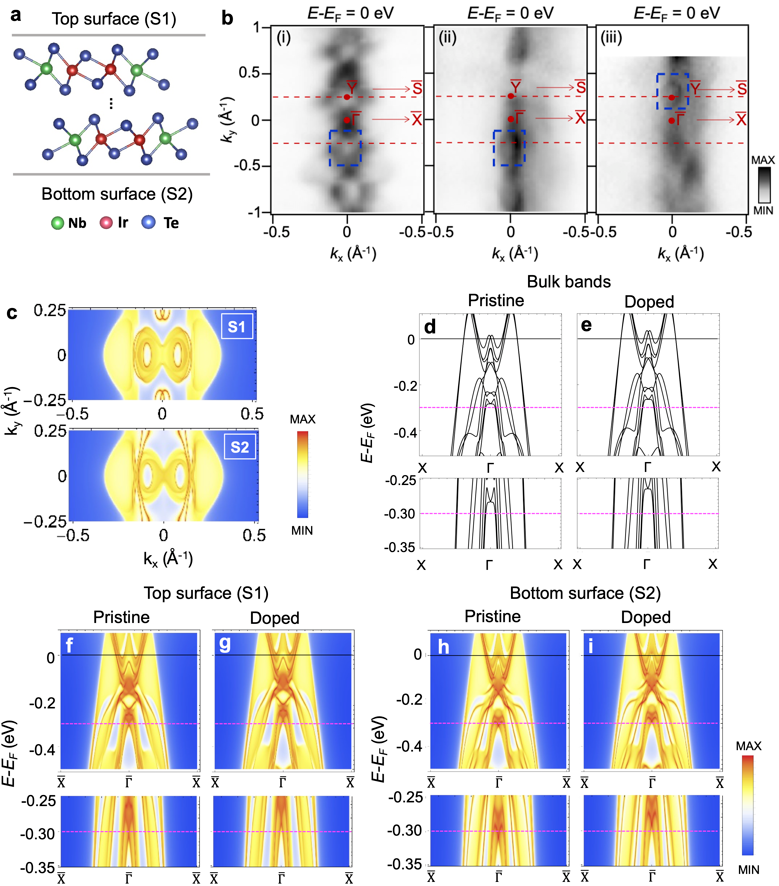


Supplementary Fig. 11. Sample-dependent ARPES and surface band structures. a. Schematics of the thin-film geometry showing top and bottom surfaces, denoted as S1 and S2, respectively. b. The Fermi surfaces obtained for three independent experiments (i)-(iii) using different samples, measured at 20 K, 20 K, and 10 K, respectively. The blue-dashed boxes show double hole pockets near the Y point that exist only on the S1 terminated surface. c. Fermi surfaces of surface band structures calculated for S1 (top panel) and S2 termination (bottom panel). Bulk band structures along Γ-X high symmetry line for undoped (d) and 0.025 h/f.u. doped (e) cases. The solid black and dashed magenta lines denote the Fermi energy and the binding energy of -0.3 eV. f-i. Surface band structures along $\overline{\Gamma}$-$\overline{X}$ high symmetry line: (f) undoped with S1 termination, (g) 0.025 h/f.u. doped with S1 termination, (h) undoped with S2 termination, (i) 0.025 h/f.u. doped with S2 termination. The bottom panels of (d-i) show band structures around the -0.3 eV.

We have examined the surface termination for the ARPES measurements based on the surface-dependent features of the Fermi surface. There are two different top and bottom surface terminations in NbIrTe_4_ as shown in Supplementary Fig. 11a denoted by S1 and S2, respectively, following the definition of the previous report^4^. We measure Fermi surfaces for three different samples as shown in Supplementary Fig. 11**b** and find that all the samples show elliptical hole pockets at the Y point. To identify surface-dependent features in the ARPES data, we perform surface band structure calculations (panel **c**) that can be considered simulated ARPES measurements and find that only the S1 terminate surface has hole pockets at Y point, consistent with the Fermi surface in the previous report^4^. Thus, the comparison between measured and simulated ARPES spectra suggests that the surface of our samples shows the characteristics of the S1 termination.

In addition to identifying the surface termination, we check whether there are surface-dependent changes in the band structures induced by the chemical potential shift using theoretical calculations. The panels (**d-i)** in Supplementary Fig. 11 present the bulk and surface band structures for undoped and hole-doped NbIrTe_4_, in which the hole-doping effect is included by reducing the number of electrons (0.025 h/f.u.) with a compensating uniform background charge. The amount of doping is chosen such that the chemical potential shift of the bulk band structures is about 20 meV, close to the estimated value from ARPES (panels **d** and **e**).

We find that the surface band structures along the $\overline{\boldsymbol{\Gamma}}$**-**$\overline{\mathbf{X}}$ high-symmetry line for both S1 and S2 terminations exhibit uniform upward shifts with amounts almost identical to the bulk bands. Thus, the temperature-dependent shift around -0.3 eV that we identify with ARPES spectra can be interpreted as the approximately same chemical potential shift, regardless of the surface termination.

**Supplementary Note 12. Doping-dependent electronic structures for the slab geometry**


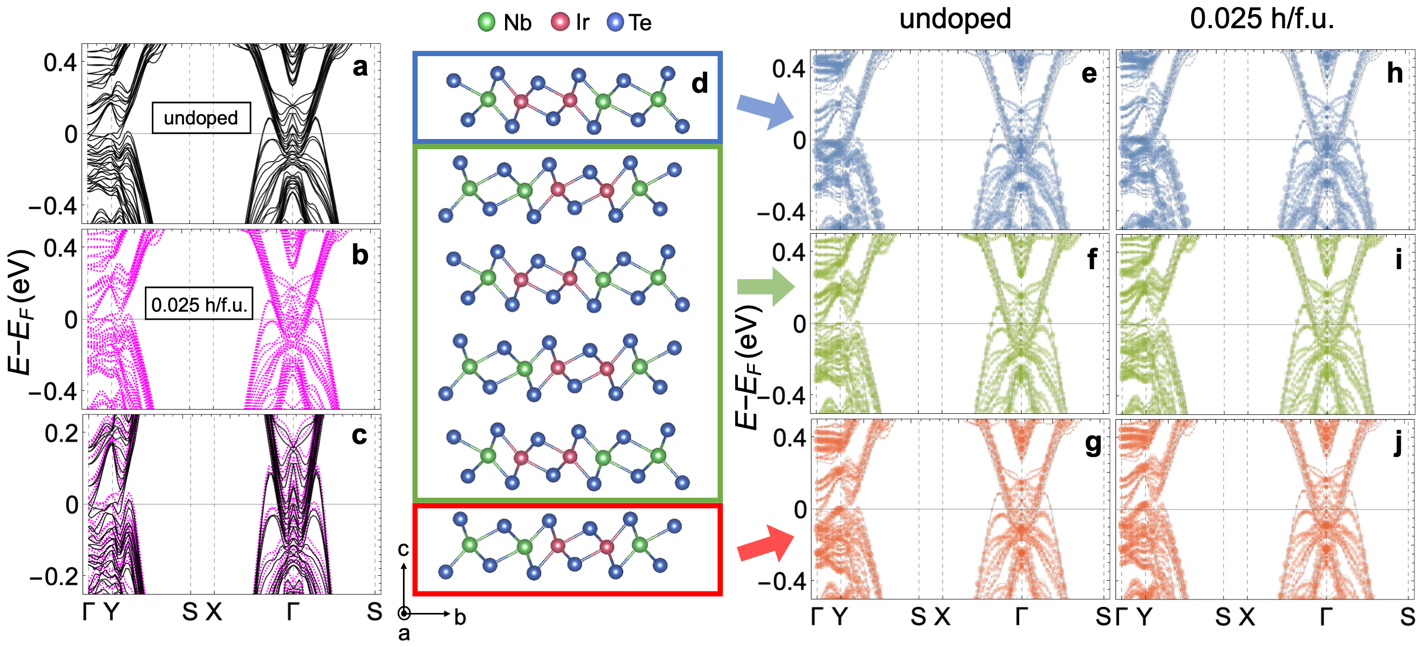


**Supplementary Fig. 12.** **Electronic structures of six-layer slab geometry for undoped and 0.025 h/f.u. doped NbIrTe_4_.** Slab band structures of (**a**) undoped and (**b**) doped NbIrTe_4_. **c**. Comparison between the undoped (black lines) and doped (magenta lines) band dispersions around the Fermi energy. **d**. Atomic configuration of the six-layer slab. The blue, green, and red boxed regions represent the top surface layer, middle layers, and bottom surface layer, respectively. **e**-**g**. Band structures of the undoped slab projected on the atoms in the top surface layer (panel **e**), middle layers (panel **f)**, and bottom surface layer (panel **g**). **h**-**j**. Band structures of the doped slab projected on the atoms in the top surface layer (panel **h**), middle layers (panel **i**), and bottom surface layer (panel **j**).

We calculate the electronic structures of the six-layer slab with undoped and doped (0.025 h/f.u.) cases. Supplementary Fig. 12 (a) and (b) present the band structures of the undoped and doped six-layer slab geometry, respectively. We find that the effect of doping is mainly the rigid band shift of about 20 meV as shown in the comparison around the Fermi energy (panel (c)), which is consistent with our bulk calculations.

In addition, we evaluate the projected bands on the atoms in the top and bottom layers and their doping dependence, since surface regions lacking the glide mirror symmetry ({*M_b_*|(1/2,0,1/2)}) of the bulk structure mainly contribute to BCD^2^. Supplementary Fig. 12 (d) shows the atomic configuration of the six-layer slab, where we divide the atomic structure into the top surface layer, middle bulk-like layers, and bottom surface layer, shown in blue, green, and red boxes, respectively. Supplementary Fig. 12 (e), (f), and (g) are bands projected on the atoms belonging to the top, middle, and bottom layers, respectively, and similarly, Supplementary Fig. 12 (h-j) are corresponding atom-projected bands for the doped case. We find no significant differences between the projected bands among the three regions, consistent with the relatively weak inter-layer van der Waals coupling. More importantly, we find no noticeable changes in the projected bands with respect to doping, suggesting that the main effect of doping is a rigid shift in the band structures.

**Supplementary References**

1. Sodemann, I. and Fu, L. Quantum Nonlinear Hall Effect Induced by Berry Curvature Dipole in Time-Reversal Invariant Materials. *Phys. Rev. Lett.* **115**, 216806 (2015).

2. Kumar, D.*, et al.* Room-temperature nonlinear Hall effect and wireless radiofrequency rectification in Weyl semimetal TaIrTe_4_. *Nat. Nanotech.* **16**, 421-425 (2021).

3. Ma, Q., *et al*. Observation of the nonlinear Hall effect under time-reversal-symmetric conditions. *Nature* **565**, 337-342 (2019).

4. Ekahana, S. A., *et al*. Topological Lifshitz transition of the intersurface Fermi-arc loop in NbIrTe_4_. *Phys. Rev. B* **102**, 085126 (2020).
